# Estimation of Intravenous Drug Users’ Population in Kermanshah City, West of Iran in 2016 using Capture-recapture Method

**Published:** 2017-08-07

**Authors:** Forod Azhdar, Nader Esmaeilnasab, Ghobad Moradi, Daem Roshani, Ebrahim Ghaderi, Bijan Nori

**Affiliations:** ^1^ Social Determinants of Health Research Center, Kurdistan University of Medical Sciences, Sanandaj, Iran

**Keywords:** Capture-recapture, Intravenous Drug Users, Iran

## Abstract

**Background:** Drug abuse, particularly intravenous drug use, is one of the most common challenges in human communities so that its negative impact on economic and cultural conditions of society and physical as well as mental health of individuals is evident. We aimed to estimate the IDUs’ population in Kermanshah City, West of Iran using Capture-recapture method.

**Study Design:** A Cross-sectional study.

**Methods:** The data in this study were collected from three different sources: Drop in Centers (DICs), Out Reach Teams (ORTs) and Methadone Maintenance Treatment centers (MMTs) in Kermanshah City from Mar 2015 until Mar 2016, and then indirect Capture-recapture was used to estimate the IDUs’ population.

**Results:** The number of IDUs registered in DICs, ORTs, and MMTs were 694, 731, and 156 cases, respectively. Having determined the commonalities and removing duplicates, the number of drug users registered were 1,375 cases, after analysis of data, the number of drug users not registered in any center was estimated as 2,042 (95% CI: 1708, 2444). By counting 1,375 cases recorded in these sources, the total number of injection drug users in the Kermanshah City was about 3,417 people, (95% CI: (3083, 3819).

**Conclusions:** The prevalence of IDUs in Kermanshah City is high, which could cause severe economic and social problems in the society. To reduce the negative effects of drug use, awareness and measuring of the drug users population, seem to be necessary overtime.

## Introduction

Addiction, as a main problem in human communities can lead to loss of social capital ^1^. Drug abuse cause the happening of numerous physical and mental problems ^2^.

Substance dependency is an illness that can affect all individuals of any age, sex or race, regardless of rich or poor^3^. More than 26 million people lose their lives due to substance abuse each year and it has been predicted that this figure will amount to 40 million people per year until the next 20 years^4^. The prevalence of Drug users in Iran is probably 8.2% in the 15-64-yr-old population^2^.

Among the various forms of drug use, the IDU has taken a daily growth as a health problem in recent years. From among the population of drug users, approximately 15.9 million people take narcotic drugs through injection^5^.

Injecting drug use behaviors are known as one of the most responsible routes for HIV transmission^6^. About 3 million drug injectors are infected with HIV^5^. The prevalence of drug user in various provinces of Iran fluctuates from 2.5% in Tehran Province to 17% in Hormozgan Province^7^. In addition, the estimation of the population of IDUs and the identification of their residential place are necessary in order to provide them with appropriate services as per the recommendations of WHO^8^.

The reality is that access to accurate statistics on the population of IDUs is a very complex and difficult task because of the secrecy and constraints in contacting them. In fact, the statistics are usually obtained through the review of the data recorded in different resources and studies or through the review of epidemiological surveys^9^.

Several statistical methods have come into existence for estimating "hidden populations" based on the data from different sources. One of these methods is Capture-recapture used for the estimation of hard-to-reach populations^10^. When the exact size of a population in certain groups is required, and access to the actual number of that group is difficult, and there is no possibility of conducting a complete census, the Capture-recapture method can be used ^11^.

Activists and policy-makers in the field of addiction need the pieces of research that can provide them with a vision of the current situation in a short time in order to design effective programs. To this end, the awareness of the population of IDUs is required. Accordingly, due to the absence of an optimal surveillance system and a lack of real figures on the size of this group, this study was an attempt to estimate the population of IDUs in the Kermanshah City in order to improve and develop research activities, plan relevant policies implement and develop operational programs. Hence, this study is expected to act as a step for the development and implementation of various control programs in addition to the determination of the frequency of individuals involved in IDUs.

## Methods

This cross-sectional study was carried out using an indirect Capture-recapture method using the information recorded in three sources, namely the centers for Drop in Centers (DICs), Out Reach Teams (ORTs), and Methadone Maintenance Treatment centers (MMTs) in order to estimate the population of IDU in the Kermanshah City, West of Iran from Mar 2015 until the Mar 2016. All people of any age and sex with at least one drug injection history recorded in the office of one or more DICs, ORTs or MMTs centers were enrolled.

The study was approved by Ethics Committee of the university under the license number of IR.MUK.REC.1395/160.

In the first stage, the statistical population, time, and area coverage of the research were specified. For data collection, three sources were used. These sources included 5 centers for DICs, 14 ORTs, and 96 centers for MMTs. In total, 115 centers were used. For data collection and counting the members in each source, it was acted according to the determined checklist and the records of the registration offices, including the full name, age, gender, residential place, education level, type of the injecting drug, and marital status. After the completion of data collection, the commonalities of the different sources were determined.

When three sources, as in this study, are used for data collection, 8 subgroups are achieved as follows based on the frequency of observations in each source and the commonalities of different sources. Below, the frequency of seven groups is determined and that of one group is unclear (Table 1).

**Table 1:** Subgroups of using three source

**Subgroups**	**DICs**	**ORTs**	**MMTs**
A	1	0	0
B	0	1	0
C	0	0	1
A∩B	1	1	0
A∩C	1	0	1
B∩C	0	1	1
A∩B∩C	1	1	1
X	0	0	0

A: The number of registered cases in DICs after eliminating the commonalities B: The number of registered cases in ORTs after eliminating the commonalities C: The number of registered cases in MMTs after eliminating the commonalities A∩B: The number of registered cases between DICs and ORTs A∩C: The number of registered cases between DICs and MMTs B∩C: The number of registered cases in ORTs and MMTs A∩B∩C: The number of registered cases in three resources X: The number of cases who are not registered in any of the resources

In the next stage, the number of observations in each of these subgroups was entered into STATA-11 and different models were fitted using log-linear models and Poisson distribution. The frequency of the eighth column whose value was unknown was estimated to use each of these models. Among these 8 models, one model lacked any interactive effect, 3 models had one interactive effect, 3 models had two interactive effects, and one model had three interactive effects.

One of the most important issues that should be considered in Capture-recapture studies is the selection of the suitable model. In this regard, there are different solutions. For the selection of the appropriate model and the best estimate, it is possible to use the degree of freedom, the deviance values, Akaike Information Criterion (AIC) and Bayesian Information Criterion (BIC) criteria,^12^.

In the present study, AIC criteria have been used with the lowest values to select the appropriate model.

## Results

The total number of IDUs registered in the sources of the study amounted to 1,375 individuals by considering at least once referral. The mean value of the population's age was 34.78±8.62 yr, the population's duration of drug use was nearly 7.48±3.64 yr, and the population's mean onset of injection was equal to 27.22±7.38 yr (Table 2).

**Table 2:** Distribution of the demographics characteristics of total registered IDUs in general

**Characteristics**	**Mean**	**SD**	**Min**	**Max**
Age (yr)	34.78	8.62	17	65
Time consumption (yr)	7.48	3.64	1	18
First consumption (yr)	27.22	7.38	16	61

Male addicts constituted the highest population of drug users in the three centers (91.6% in DICs, 85% in ORTs, and 93.6% in MMTs) and the 30- 39-yr-old group was the largest age group who were living in urban areas (Table 3).

**Table 3:** Distribution of the demographics characteristics of registered IDUs by each center

**Characteristics**	**DIC**_S_		**ORT**_S_		**MMT**_S_	
	**Number**	**Percent**	**Number**	**Percent**	**Number**	**Percent**
Gender						
Male	636	91.6	621	85.0	146	93.6
Female	58	8.4	110	15.0	10	6.4
Age group (yr)				
<30	210	30.3	218	29.8	56	25.9
30-39	291	41.9	294	40.2	79	50.6
40-49	149	21.5	176	24.1	18	11.5
≥50	44	6.3	43	5.9	3	1.9
Marital status						
Single	428	61.7	528	73.3	98	62.8
Married	195	28.1	133	18.2	46	29.5
Divorced	63	9.1	66	9.0	11	7.1
Widow	8	1.2	4	0.5	1	0.6
Region						
Urban	658	94.8	692	94.7	148	94.5
Rural	36	5.2	39	5.3	8	5.1

**Figure 1:**
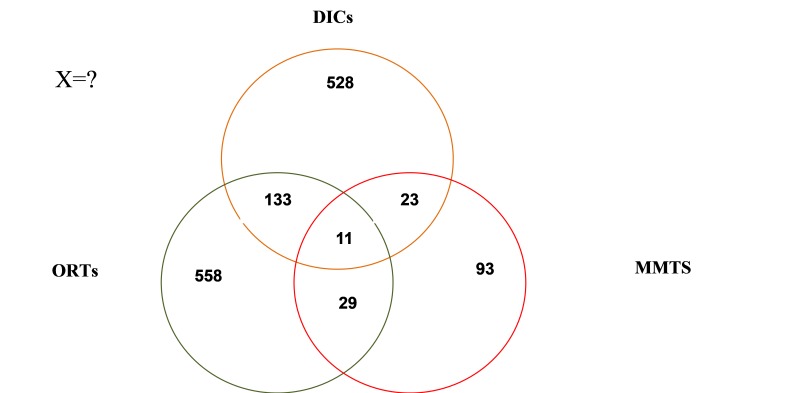
Distribution of IDUs registered Drop in Centers (DICs), Out Reach Teams (ORTs) and Methadone Maintenance Treatment centers (MMTs) and commonalities between resources

The total number of people registered in DICs, ORTs, and MMTs equaled 694, 731, and 156 cases, respectively. From this population, the number of 133 individuals was subscribed to DICs and ORTs at the same time; the number of 23 individuals was subscribed to DICs and MMTs at the same time; the number of 29 individuals was subscribed to ORTs and MMTs at the same time; and the number of 11 individuals was jointly subscribed to all the three centers. The number of people in each source after deleting the duplicates, as well as the number of people subscribed to more than one source, is shown in Figure 1.

After statistical data analysis, 8 different models according to the Table 4 were obtained. Eventually, out of the eight models, model No. 1 (A B C) with the criteria of AIC = -2.54 and BIC = -2.39 was considered as the best and most appropriate model for the estimation of the unregistered cases and the total number of injecting drug users. Accordingly, the population of IDUs who had not been registered in any of the three sources was estimated an approximate of 2,042 (95% CI: 1708, 2444). Therefore, while taking into account the 1,375 people who had been registered in different sources, it was predicted that the total number of IDUs in the mentioned city would be equal to 3,417 (95%CI: 3083, 3819) (Table 4).

**Table 4:** Log linear models fitted to three source of IDUs, unregistered and total estimated number of them

**Model**	**K**	**df**	**Log likelihood**	**G2**	***P*** ** value**	**X**	**CI 95%**	**N**	**CI 95%**	**AIC**	**BIC**
A B C	3	3	-23.63	3.46	0.33	2042	1708, 2444	3417	3083, 3819	-2.54	-2.38
A B C AB	4	2	-23.23	2.65	0.27	1799	1298, 2504	3174	2673, 3879	-1.35	-1.24
A B C AC	4	2	-23.51	3.21	0.20	2077	1715, 2520	3452	3090, 3895	-0.79	-0.68
A B C BC	4	2	-22.65	1.50	0.47	2150	1772, 2614	3525	3147, 3989	-2.50	-2.39
A B C AB AC	5	1	-23.23	2.65	0.10	1789	1180, 2786	3146	2555, 4166	0.65	0.70
A B C AB BC	5	1	-22.65	1.20	0.22	2135	1362, 3476	3510	2737, 4851	-0.50	-0.44
A B C AC BC	5	1	-22.40	0.99	0.32	2215	1796, 2743	3590	3171, 4118	-1.01	-0.95
A B C AB AC BC	6	0	-21.90	0	1	3398	1408, 7899	4773	2783, 9274	0	0

K: number of parameters; df: degree of freedom; G^2^: deviance; X: unregistered number; CI confidence interval; N: total number; AIC: Akaike Information Criterion; BIC: Bayesian Information Criterion.

The identified percentage combined in the three sources was nearly 46% where the source of ORTs took up the highest percentage of identification (21.3%) and MMTs had the lowest percentage of identification (4.5%).

## Discussion

The findings of this study revealed that the prevalence of IDUs is about 0.43% in the adult population at the city of Kermanshah; therefore, this rate can prepare the ground for the development of contagious diseases via blood, including AIDS and hepatitis B and C. The harmful consequences of addiction not only involves the sufferer him/herself but also entangles the spouse, children, and the whole community at large and puts social norms and values at risk ^13^. IDU has a negative impact on economic and social conditions and causes physical illnesses, and mental disorders and events^14, 15^.

Our results indicate that the majority of IDUs are not covered by any service providing centers; therefore, planning and implementation of necessary measures are required to identify this group of people. In this study, the indirect Capture-recapture method was used for the statistical estimation of IDUs. Although the use of statistical methods for estimating hidden and hard-to-reach populations is available, practical, and useful, care and discretion should be exercised in the presentation and interpretation of results with regard to the assumptions and limitations like other statistical methods.

Since registered information and data available in various sources are used in indirect Capture-recapture studies, the number of used information sources can affect the validity of the results. If two information sources are used, the sources should have a high overlap level to provide reliable results. If the number of used information sources is more than two, the overlap of the sources will increase and the results will be closer to reality. Usually, the best estimate is made when 3 to 5 sources are used^11^. In this study, three sources of information registration were used for data collection so that the results would enjoy greater reliability.

In recent years, the employment of Capture-recapture method for estimating hidden populations has become more prevalent among researchers. In Montreal Iceland, the Capture-recapture method with 6 information sources was used to estimate the population of IDUs. The number of registered people in 6 sources was 1,480 while 278 people overlapped between different sources. Indeed, 4 individuals were subscribed to 4 sources, 61 people were subscribed to 3 sources, 213 people shared two sources, and the number of intravenous drug users was estimated at around 3,910 persons^9^.

In Iran, percussion technique was used to estimate the total population of drug users. The information of the people who had referred to the laboratory for addiction tests and other specific purposes, such as employment, marriage, driving licenses, and business licenses was collected. It was estimated that the number of drug users in Iran was about 1 million^16^.

In Tehran, Iran for estimating the female sex worker population (FSWs), Direct Capture-recapture was used and the population of this group was estimated as 690 people (%95 CI: 633, 747)^17^.

In the same way, Khazaea et al. estimated the population of IDUs in the city of Hamadan through Capture-recapture method. They used the recorded information in three sources where the number of registered injecting drug users was obtained about 1,369 cases via census. In that study, the total number of IDUs was estimated equal to 11,333 cases based on the commonalities of different sources and Log-linear models^5^.

The comparison of the results of the present study with those of some other studies mentioned earlier carried out with this method in certain groups suggests that the surveillance and information systems in the field of addiction are incomplete and deficient in many countries, including Iran and the presented statistics are much lower than the actual population of this group of people.

The limitation of the study was as follows. Since no identification certificate was received from the clients, it is possible that people would have used different names to introduce themselves when referring to different centers, which might have led to overestimation. To solve this problem, one personnel (with sufficient knowledge about the clients) helped with the completion of the checklist.

## Conclusions

As regards the relatively high prevalence of injecting drug use and the low percentage of identification in individuals by service providers, the following measures should be taken in this regard: development of service centers, accessibility to diagnostic facilities, the presence of harm reduction programs, and distribution of syringes free of charge. With respect to the existing deficiencies in the surveillance system, such estimations and measures should be repeated over time in the epidemiology of drug use.

## Acknowledgment

We express our thankful regards to the head of Kermanshah Health Center; the officials of Drop in Centers, the authorities in Methadone Maintenance Treatment Centers, Out Reach teams' staff, and other participants in the study.

## Conflict of interest statement

None.

## Funding

This article is derived from a master's thesis in the field of Epidemiology at the Kurdistan University of Medical Sciences and Health Services.

## Highlights

- The total number of IDUs registered in the sources of this study was 1,375 people
- The IDUs number unregistered in any center is estimated 2042.
- The total number of IDUs was estimated nearly 3417 people.
- The mean value of the population's age equaled 34.78±8.62 years
- The IDUs prevalence in Kermanshah City is about 0.43% in 15-64 year-old population.
